# A novel 3D nanofibre scaffold conserves the plasticity of glioblastoma stem cell invasion by regulating galectin-3 and integrin-β1 expression

**DOI:** 10.1038/s41598-019-51108-w

**Published:** 2019-10-10

**Authors:** Ali Saleh, Emilie Marhuenda, Christine Fabre, Zahra Hassani, Jan de Weille, Hassan Boukhaddaoui, Sophie Guelfi, Igor Lima Maldonado, Jean- Philippe Hugnot, Hugues Duffau, Luc Bauchet, David Cornu, Norbert Bakalara

**Affiliations:** 1 Institut des Neurosciences de Montpellier, INM, U-1051, Univ. Montpellier, CHU de Montpellier, ENSCM, INSERM, Montpellier, France; 2 UMR 1253, iBrain, Univ. Tours, Inserm, Tours, CHRU de Tours, Le Studium Loire Valley Institute for Advanced Studies, Montpellier, France; 3 Institut Européen des Membranes, IEM, UMR-5635, Univ. Montpellier, ENSCM, CNRS, Montpellier, France

**Keywords:** CNS cancer, Focal adhesion

## Abstract

Glioblastoma Multiforme (GBM) invasiveness renders complete surgical resection impossible and highly invasive Glioblastoma Initiating Cells (GICs) are responsible for tumour recurrence. Their dissemination occurs along pre-existing fibrillary brain structures comprising the aligned myelinated fibres of the corpus callosum (CC) and the laminin (LN)-rich basal lamina of blood vessels. The extracellular matrix (ECM) of these environments regulates GIC migration, but the underlying mechanisms remain largely unknown. In order to recapitulate the composition and the topographic properties of the cerebral ECM in the migration of GICs, we have set up a new aligned polyacrylonitrile (PAN)-derived nanofiber (NF) scaffold. This system is suitable for drug screening as well as discrimination of the migration potential of different glioblastoma stem cells. Functionalisation with LN increases the spatial anisotropy of migration and modulates its mode from collective to single cell migration. Mechanistically, equally similar to what has been observed for mesenchymal migration of GBM *in vivo*, is the upregulation of galectin-3 and integrin-β1 in Gli4 cells migrating on our NF scaffold. Downregulation of Calpain-2 in GICs migrating *in vivo* along the CC and *in vitro* on LN-coated NF underlines a difference in the turnover of focal adhesion (FA) molecules between single-cell and collective types of migration.

## Introduction

In the last few years accumulating evidence pointed to the ECM in shaping cancer traits. The biophysical and biochemical cues stemming from the ECM affect tumour growth, proliferation, apoptosis, angiogenesis, invasion and metastasis^1^. Recent data unequivocally demonstrated that malignant cellular modifications are intricately linked to the surrounding microenvironment, making traditional bidimensional cell culture models deficient. For instance, 2D models do not reproduce the *in vivo* conditions of surface nanotopography, stiffness, or polarity^2,3^. This could explain the discrepancies observed between *in vitro* studies and pre-clinical trials during drug development^4^. Moreover, stress fibres and FA are significantly reduced in 3D configurations, whereas cellular deformation, a limiting process in 3D migration, is not essential in 2D^5^. Therefore, different tridimensional culture models have been established to overcome these limitations such as hydrogels, sponges, decellularized tissues or cell layers and fibres^6–9^. Electrospun NF in particular are emerging in cancer research^10^. Nevertheless, some experimental obstacles remain in these systems as for instance the unspecified composition of commercial matrix, poor mechanical properties, requirement to include cells before gelation, difficulty of creating a stable and controllable macroporosity to obtain cell confinement and the impossibility of creating a spatially anisotropic microenvironment with a constant chemical composition (hydrogels), a poor cellular infiltration or restricted ingrowth and cytotoxicity (fibres)^3^. Besides that, the possibility to carry out omics analysis and large-scale extraction of proteins and RNAs without degradation of the substrate would be highly desirable.

Glioblastoma multiforme (GBM) is a highly invasive primary brain tumour. GICs that penetrate the subarachnoid space or intravasate into the cerebral microvasculature are chemo- and radio-resistant and hinder complete surgical resection^11^. A critical process for GIC invasion is the ECM remodelling. GICs take advantage of the combination of multiple molecular and physical mechanisms along pre-existing tracks of least resistance such as the white matter which guides and facilitates their invasive behaviour^12^. GICs use a mesenchymal single cell migration mode to migrate away from the main tumour bulk^13^ which is characteristic of disseminating glioma^14^. In addition, they may form *in vivo* multicellular networks or clusters implicated in their invasive capacity and radioresistance^15,16^. To recapitulate these different migration modes and to mimic the topography of the white matter tracts the biochemical composition of the brain ECM, we developed new NF scaffolds of aligned (aNF) and non-aligned (naNF) of stabilized PAN, which are either partially functionalized with LN (+LN) or not (−LN). Taking advantage of the diversity of its functional groups after stabilization/oxidation and of its tuneable mechanical properties, we propose a new application of PAN, which can challenge biopolymers in the biomedical fields. We explored how the topography and biochemical components of the NF influence glioma haptotaxis and haptokinesis. We correlated our results with *in vivo* xenografts of human GIC into the brain of nude mice.

## Results

### NF network production and physical characterization

**Figure 1:**
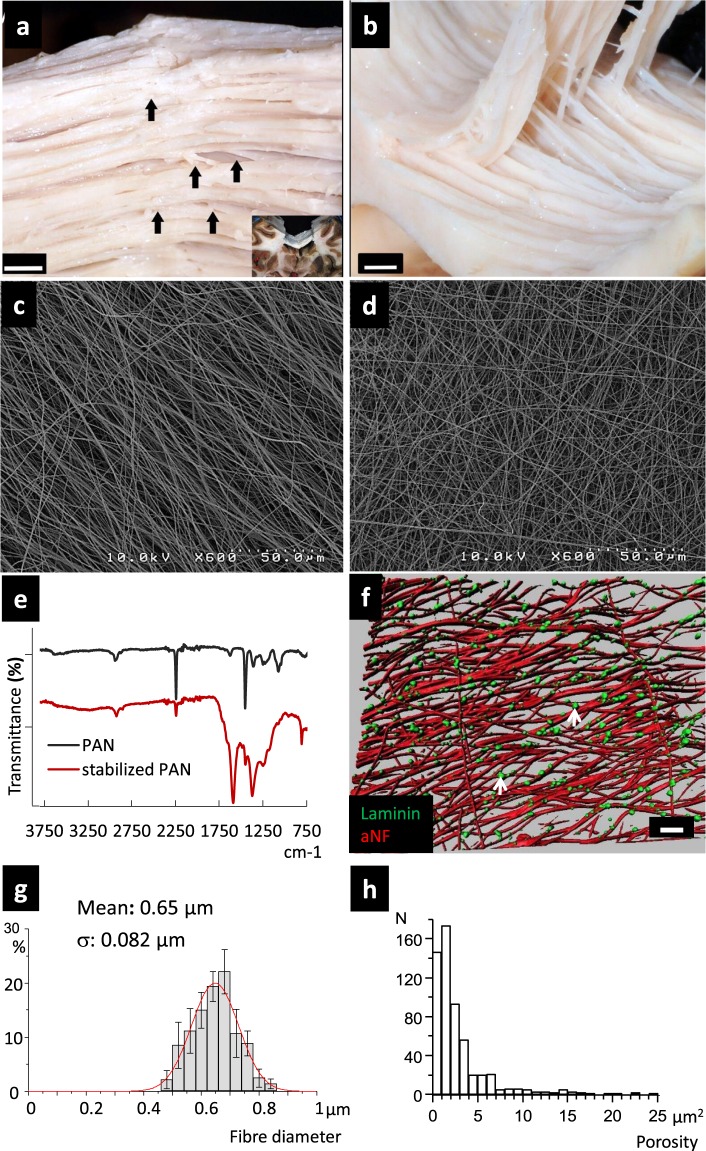
Physical characterization of NF and their functionalization with LN. (**a**) Upper posterior view of the dorsal aspect of the trunk of the CC prepared with a variant of the Klinger’s Technique for macroscopic fibre dissection (specimen in the box). (**b**) Antero-superior view. Appearance of the CC in the course of delamination in the lateral-lateral direction (same specimen as in A). (**c,d**) PAN NF were produced by electrospinning from DMF solution. The thermal treatment was conducted in air at atmospheric pressure in a chamber furnace. During this step, stabilization and oxidation occurred yielding an infusible mat which turned from white to brown. Scanning Electron Microscopy images showing the alignment of the aNF (**c**) and naNF (**d**). The aNF network corresponds more to a preferential orientation than to strictly parallel fibres. (**e**) FTIR spectra of PAN and stabilized PAN emphasizing the chemical structure modification during the thermal treatment (Wavenumber in cm^−1^). (**f**) Image reconstitution corresponding to z-stack images of LN immunostaining on aNF. LN deposits (green) are discontinuously distributed on the NF (white arrows). The NFs are autofluorescent and appear in red (false colour red) (scale bar = 20 µm). (**g**) Distribution of the NF diameters. The histogram was fitted with a single Gaussian function shown in red. (**h**) Distribution of the scaffold porosity.

The CC is the favourite route to the contralateral hemisphere of glioblastoma cells^17^. Figure 1a,b highlight the three-dimensional anatomic organization of the heterotypic fibres in the trunk of the CC. To better understand, characterize and target migrating glioblastoma cells on the CC, we designed a NF network which could be made of aligned or non-aligned fibres (Fig. 1c,d). The purpose of this model is to be able to study the impact of the spatial and mechanical properties of a fibrous microenvironment. PAN NF have been selected for their biocompatibility and resistance to biodegradation that would interfere with a mechanistic study. Moreover, the spatial design and mechanical properties of PAN NF are easily tuneable. Fourier transformed infrared (FTIR) spectroscopy (Fig. 1e) was used to discriminate the functional groups of the stabilized PAN^18^. Commercial PAN contains traces of (free) water (3622 and 1626 cm^−1^) and bands at 2940 cm^−1^ (CH_2_, CH stretching), 2242 cm^−1^ (nitrile groups), 1453 cm^−1^ (δCH_2_), 1356 cm^−1^ (CH bending), 1249 cm^−1^ (χ CH_2_) and 1072 cm^−1^ (C-C stretching). After stabilization and oxidation, the spectrum shows a strong reduction of the nitrile band at 2242 cm^−1^, broad weak bands from 3100 to 3600 cm^−1^ attributed to OH and NH stretching, a very strong band at 1589 cm^−1^ (C = C stretching and NH bending), a strong band at 1372 cm^−1^ (NH and CH bending) and a weak band at 807 cm^−1^ (C = C-H rocking in the aromatic plane). When subjected to UV light, NFs appear fluorescent. They emit in red, green and at a much lower extent in blue and infrared (data not shown). The NFs were decorated with LN. Spots of LN deposit can be seen discontinuously distributed along the NF + LN (Fig. 1f, white arrows). The diameter of the NF of 0.65 ± 0.082 µM (Fig. 1g) is similar to the diameter of the axons in the CC (0.64 ± 0.42 µm)^19^. The porosity of the aNF scaffold predominantly ranges between 0.5 µm² and 7 µm² (Fig. 1h). This creates a confinement situation which is required to trigger linear glioma migration.

### The NFs constitute a suitable tridimensional environment for GIC adhesion and migration

**Figure 2:**
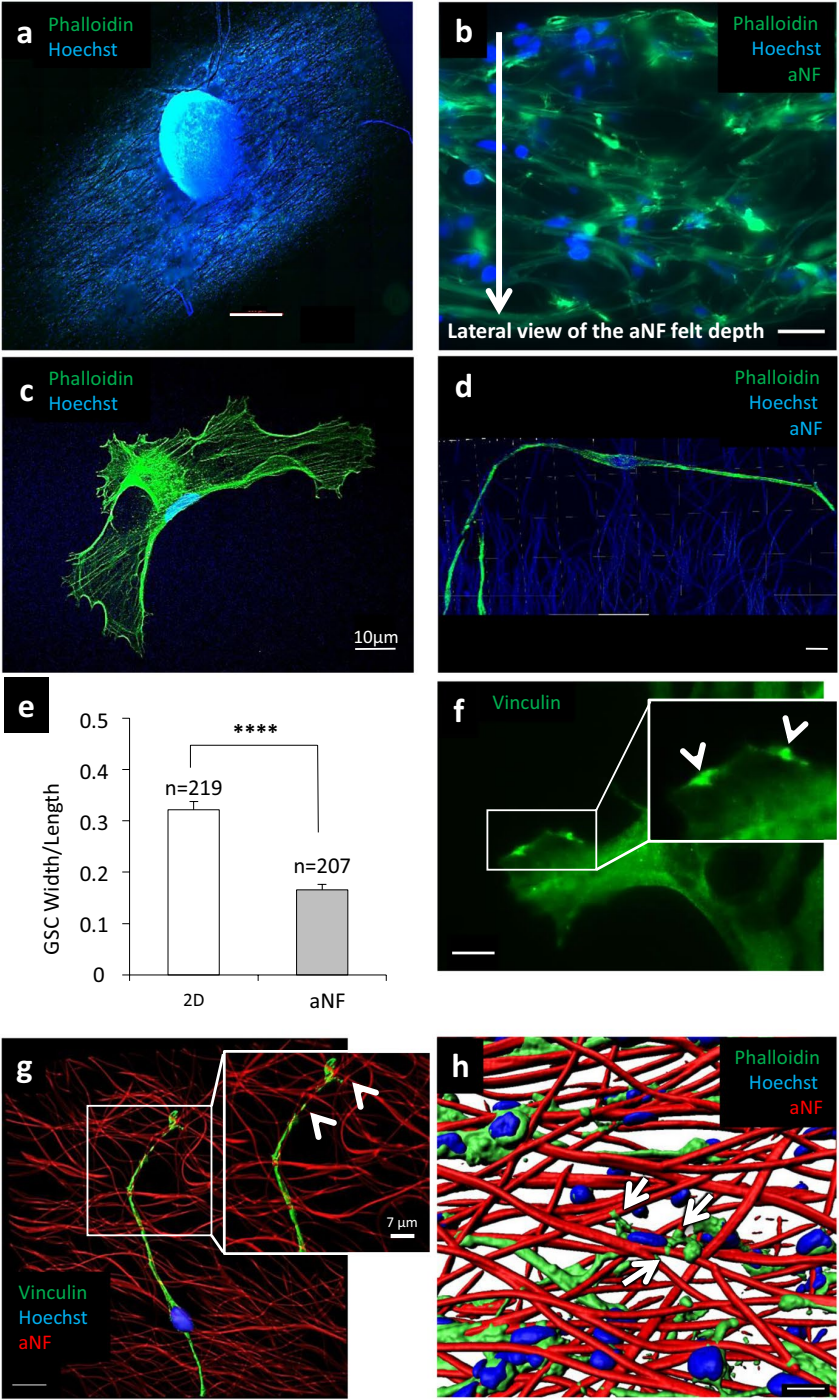
NF create a tridimensional microenvironment convenient for GIC proliferation, adhesion and migration. (**a**) Image showing the adherence and the penetration of a NS in the aNF network (scale bar = 500 µm). (**b**) A lateral view of the repartition of migrating Gli4 cells (away from the NS) deep inside the aNF network (scale bar = 20 µm). (**c,d**) Morphology of Gli4 cells on 2D (**c**) (scale bar = 10 µm) and NF. (**d**) (Z-stack 3D reconstitution, scale bar = 20 µm). The actin cytoskeleton was stained with phalloidin (green) and the nucleus with Hoechst 33342 (blue). (**e**) Quantification of Gli4 cells morphology measuring the ration width/length of cells grown on 2D compared to those grown on aNF − LN (n = 3 independent experiments). (**f**) The FA marker vinculin was used to stain Gli4 in 2D + LN (scale bar = 10 µm), white arrows indicate spots of vinculin. (**g**) Z-stack 3D reconstitution showing Vinculin (green) in Gli4 adhesion on aNF + LN. Nucleus was stained with Hoechst (blue) (scale bar = 15 µm). (**h**) Imaris reconstitution showing Gli4 protrusions encircling the fibres (yellow arrows (scale bar = 20 µm)). At the time of the seeding, the NS contained 10,000 cells. Images were captured six days after NS seeding on aNF + LN.

Gli4 cells express several typical markers of neural precursors, are multipotent and generate tumours *in vivo*^20^. When seeded on 2D in differentiation medium, neurospheres (NS) adhere to the support and cells migrate away from the NS (Fig. 2a). Analysis of the repartition of the Gli4 cells, within the fibre scaffold shows that cells migrate deeply into it (Fig. 2b). Gli4 cells feature an extensive and flat network of stress fibres when plated in 2D conditions (Fig. 2c), whereas Gli4 seeded on NF appear as elongated cells presenting filopodia while no stress fibres are visible (Fig. 2d,e). In 2D, vinculin spots localize at the leading edges of the lamellipodia (Fig. 2f). On NF, Gli4 cells send cellular extensions in various directions to attach to several fibres in a tridimensional manner (Fig. 2g,h, yellow arrows). Rather than focal, adhesion sites are spread along filopodia, which also encircle the fibres on NF (Fig. 2g,h). It has previously been reported that astrocytic brain tumour cells extend ultra-long membrane protrusions that they use as routes for brain invasion^16^. These data show that NFs constitute a pertinent permissive tridimensional microenvironment for glioblastoma cell migration and adhesion.

### Gli4 migrate individually or collectively in the presence or absence of LN on PAN NF

During glioblastoma progression, the secretion of ECM molecules such as LN, fibronectin or hyaluronic acid increase significantly^13^ and the microenvironment is modified by deposing these molecules. For this reason, we address the question whether or not LN-coated fibres modify GIC migration.

**Figure 3:**
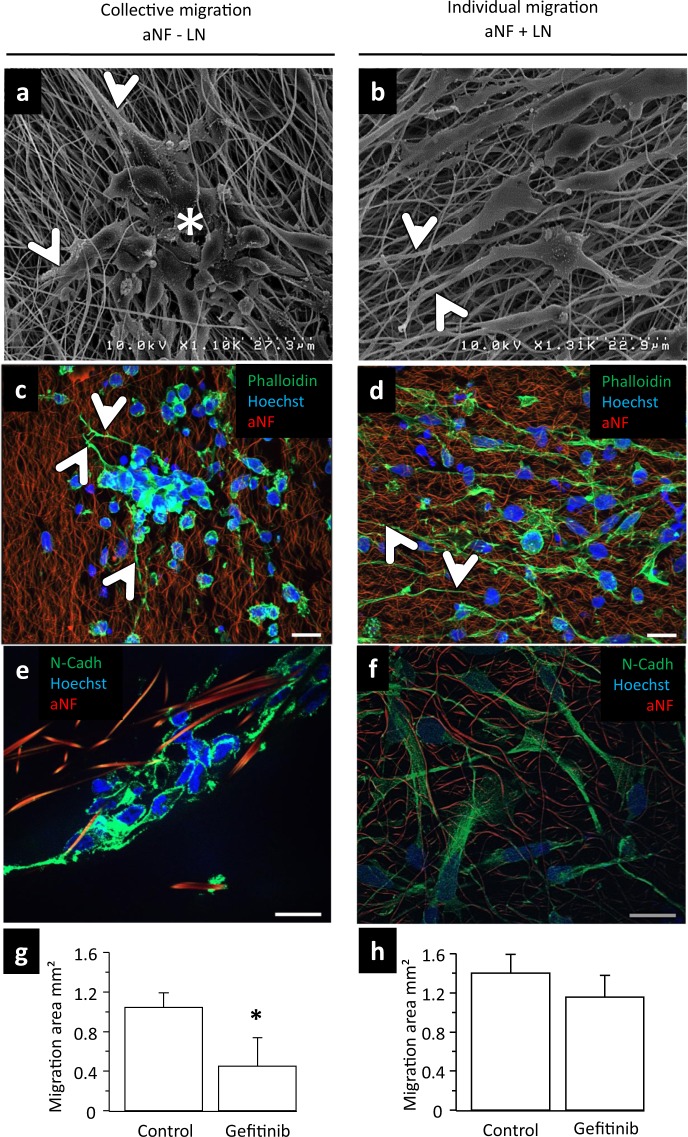
Gli4 cells migrate individually or collectively in the presence or absence of LN respectively. (**a,c,e**) The migration mode of Gli4 cells was analysed in absence of LN on aNF. (**b,d,f**) The migration mode of Gli4 cells was analysed in presence of LN on aNF. At the time of seeding, the NS contained 7500 cells. The images were captured five days after NS seeding. The aNFs are autofluorescent and appear in red the nucleus in blue (Hoechst 33342 blue). (**a,b**) The migrating Gli4 cells were visualized using scanning electron microscopy. (**c,d**) Immunofluorescence analysis of migrating Gli4. The actin cytoskeleton was stained with green phalloidin (scale bar = 20 µm). The arrowheads indicate the filopodia extensions of the peripheral cells. (**e,f**) Cell-cell junctions were stained using anti N-cadherin specific antibody (green) (scale bar = 20 µm). (**g,h**) Inhibition of migration of Gli4 in proliferation medium on non-coated (−LN) fibres (**g**) and LN coated (+LN) fibres (**h**) (n = 3). Gefitinib was used at 3 µM.

In the absence of LN, Gli4 cells migrate collectively by forming aggregates composed of tens of tightly associated cells (Fig. 3a,c,e). On the contrary, Gli4 do not aggregate on NF + LN and migrate as single cells (Fig. 3b,d,f). Cells are rounded at the centre of the cell mass (Fig. 3a, white star), while at the border they are bipolar and have a filopodial protrusion (Fig. 3a, arrowheads). Because cadherin expression and actin cytoskeleton remodelling were reported to control collective migration^21^, we analysed the organization of the actin cytoskeleton and the N-cadherin-mediated adherent junctions. On NFs, actin cytoskeleton-specific shows that most of the cells are rounded and appear to form a continuum within aggregates (Fig. 3c). Collectively migrating Gli4 express N-Cadherin at the plasma membrane (Fig. 3d). Thus N-Cadherin-mediated adherent junctions are maintained during collective Gli4 migration. On the contrary, Gli4 behave as single cells on NF + LN, they are bipolar (Fig. 3d,f, arrowheads).

Using human *ex-vivo* GBM slice cultures, it has been found that GBM tumours that have their EGFR gene amplified are more invasive than those that have not, suggesting that EGF signalling stimulates GBM cell migration. In line with this notion is that migration of this type of tumour was slowed down by the tyrosine kinase inhibitor, Gefitinib^22^. Subsequently, clinical expectations raised by this report were unfortunately not met by targeting the EGFR signalling cascade with Gefitinib^23^. Several hypotheses can be forwarded to explain the discrepancy between the *in vitro* and clinical results, one of which pertains to the experimental context. To verify the clinical relevance of our NF culture design, we tested the effect of gefitinib on migrating GICs in the presence of an EGFR stimulation background. It was found that Gefitinib is indeed without effect if GICs migrate individually on LN-coated fibres, but that is very efficient in reducing the speed of collectively migrating cells on uncoated fibres (Fig. 3g,h).

### Gli4 haptokinesis and haptotaxis result from the orientation and coating of the NF scaffold

**Figure 4:**
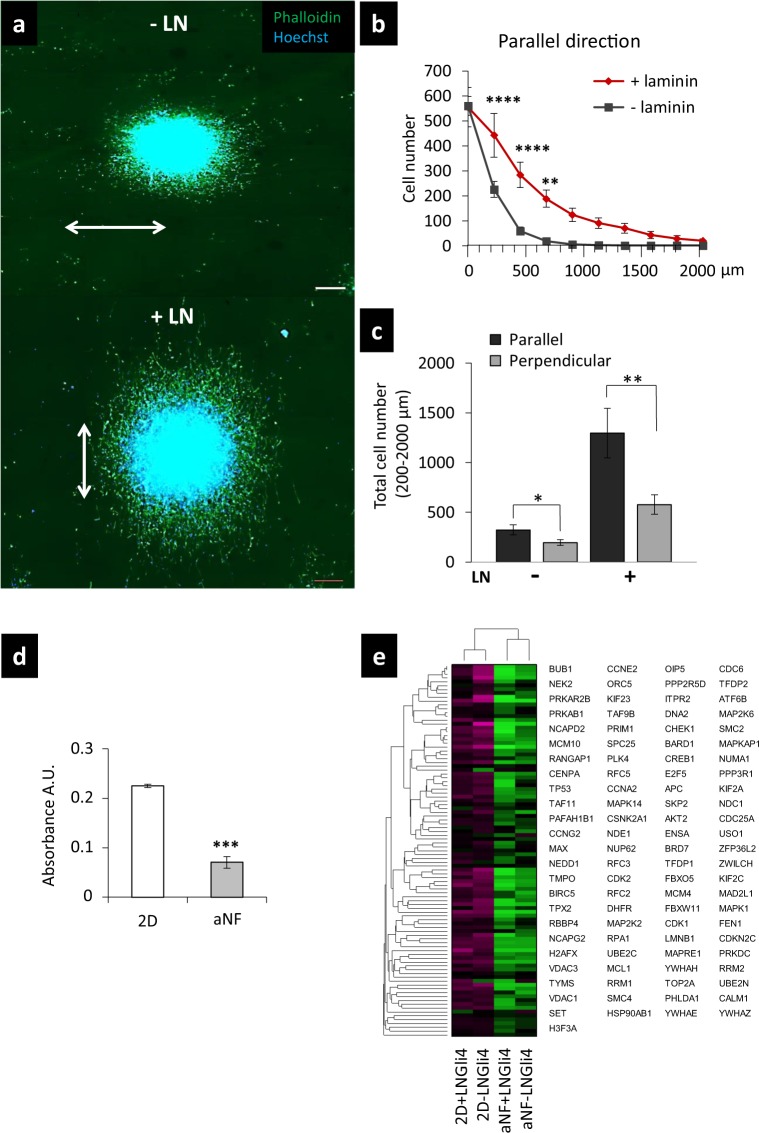
aNF microenvironment reduces the growth ratio and fibre orientation determines the direction of migration of GIC. (**a**) The NS contained 5000 cells at the beginning of each experiment at day zero. The images were taken after six days of culture in differentiation conditions. The direction of the NF. Nuclei were stained with Hoechst 33342 (blue) (scale bar = 200 µm). For distances between 0 and 200 µm from the border of the NS, LN coating does not increase the number of migrating cells at all. (**b**) Quantification of the number of Gli4 cells migrating in the direction of the aNF in presence or absence of LN, the direction of the NF. (**c**) Quantification of the total number of migrating Gli4 cells in the parallel or perpendicular direction of aNF and in the presence or absence of LN. The number of cells was counted in the area between 200 µm and 2000 µm from the border of the NS. This area was chosen in order to exclude the increase in cell number related to expansive growth near the NS. **p < 0.01 (Student’s t-test). All Data are representative of two independent experiments. The length of the migration area was demarcated between the NS border and a distance of 2 mm in the direction of migration. (**d**) Proliferation rate was estimated using an MTT test 4 days after seeding of 5000 dissociated Gli4 cells in 2D and NF. Unpaired Student’s t-test. (**e**) Heat map of the 98 genes associated with cell cycle and differentially expressed between Gli4 cells cultivated in either 2D or NF +/− LN. The list alongside the heatmap comprises ‘cell cycle’ genes that are expressed differently between the 2D and NF samples. Significance was estimated with Student’s t-test (n ≥ 4).

To investigate the impact of the ECM topography on GIC migration, we compared the direction of migration of Gli4 cells on aNF and naNF functionalized or not with LN. First of all, our results show that on aNF, Gli4 cells migrate predominantly according to the orientation of the NF (Fig. 4a). We observed a significant higher number of migrating cells in the direction parallel to the NF than in the perpendicular direction, particularly when LN is present (Fig. 4b,c).

A proliferation test indicated the lower proliferation rate of Gli4 cultured on aNF with respect to 2D (Fig. 4d). Transcriptome analysis revealed that 98 genes involved in cell cycle progression as well as members of the kinesin family were expressed by at least a factor of 1.5X less in cells grown on NF than on 2D, suggesting a shift to the invading, less proliferative mesenchymal phenotype of GIC cells in a NF context (Fig. 4e). Accordingly, the expression of mesenchymal and pre-metastatic stem cell markers such as ATXN1, ALCAM, CD9, ITGA7 and CD44 are maintained in NF vs 2D culture conditions (Supplementary Figure 1). We may conclude from this analysis that Gli4 cultured in the NF microenvironment retain their mesenchymal phenotype. Moreover, the 3.3X overexpression of CHI3L1 (Supplementary Figure 1) in NF could promote as reported for liver cancer cell migration and invasion^24^.

### Cellular adhesion to the ECM and FA dynamics of Gli4 cells differ between conventional 2D planar surfaces and aNF

**Figure 5:**
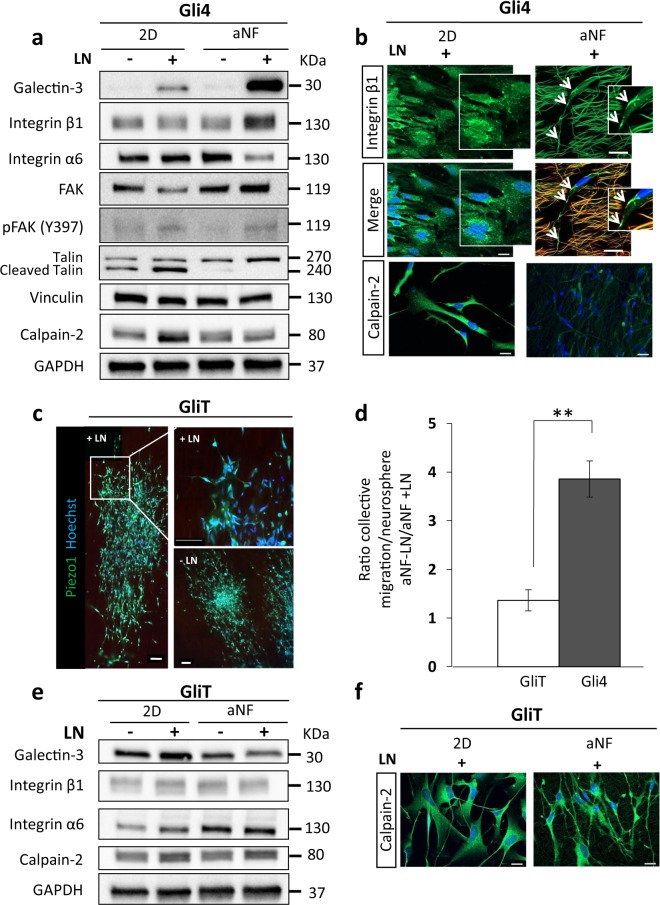
Gli4 and GliT behave differently on aNF +/− LN. (**a**) Gli4 expression of galectin-3, integrin β1, integrin α6, FAK, p-FAK (Y397), vinculin, calpain-2. (**b**) GlI4 immunostaining for integrin β1 (green) and calpain-2 (green) (scale bar = 20 µm). White arrow heads indicate the Integrin β1 co-localization with the fibres at the sites of attachment. (**c**) Although plated in presence of LN, GliT cells migrate collectively (Piezo 1 staining in green is used as a membrane marker; scale bar = 100 µm). (**d**) Quantification of collective migration between Gli4 and GliT on aNF +/− LN. (**e**) Ratio of the number of collective migration events in the absence or presence of LN on aNF for Gli4 and GliT. (**e**) GliT expression of galectin-3, integrin β1, integrin α 6, calpain-2. GAPDH was used as a loading control for protein normalization. Data are representative of three independent experiments, ****p < 0.0001, **p < 0.01 (Student’s t-test). (**f**) Immunostaining for calpain-2 (green) of GliT cells (scale bar = 20 µm). Nuclei were stained with Hoechst 33342 (blue). The aNF are faintly autofluorescent in red and green.

Important proteins implicated in migration are the multifunctional modulators of cell FA such as galectins and integrins. FAs play a central role in migration by controlling the dynamic cycles of attachment and detachment to the ECM^25^. FAs are considered as prototypical integrin-mediated cell-ECM contact sites that link cellular actin cytoskeleton to the ECM scaffold^26^. Therefore, our next aim was to compare the expression of integrin β1, α6 and galectin-3 in the different culture conditions. On aNF, the protein levels of galectin-3 and integrin β1 were respectively 6 and 2.6 fold higher in cells grown on LN-coated fibres than on non-coated fibres (Fig. 5a and Supplementary Figure 2). On 2D, only galactin-3 expression is somewhat reduced by LN coating (Fig. 5a and Supplementary Figure 2). In contrast, the protein level of integrin α6 was 2.5 times lower on LN-coated fibres whereas its level in 2D increases with LN (Fig. 5a and Supplementary Figure 2). Figure 5b shows that integrin β1 is localized at the plasma membrane of Gli4 migrating on aNF + LN (white arrowheads), while it is distributed in the cytoplasm and around the nucleus in Gli4 growing on 2D + LN. Note that on NF + LN, integrin β1 membrane staining is localized in the attachment points with the NF (Fig. 5b, white arrowheads).

According to these previous data we decided to study the expression of FA components such as calpain-2, vinculin, FAK and phosphorylation of FAK and Talin and cleaved Talin expressions in Gli4 cells on the aNF +/− LN and 2D +/− LN supports (Fig. 5a and Supplementary Figure 2). Calpain-2 expression is 2 fold higher in Gli4 cells plated on 2D + LN than in the other conditions. Immunofluorescence confirmed that calpain-2 is upregulated in 2D + LN compared to aNF + LN (Fig. 5b). The expression levels of vinculin are equal in aNF +/− LN and 2D +/− LN (Fig. 5a). Compared to 2D + LN, FAK expression increases on aNF + LN, whereas its phosphorylation ratio decreases. Talin expression and cleavage are increased in 2D + LN compared to all other conditions. We therefore conclude that FA dynamics are affected by the dimensionality and functionalization of the microenvironment.

### GliT migrate collectively irrespective of LN coating and exhibit a different pattern of expression of cell adhesion and signalling proteins compared to Gli4 cells

We examined a second primary glioblastoma cell line with a different signature, GliT. Transcriptome analysis comparing Gli4 and GliT in NS culture yielded 784 genes differentially expressed by at least a factor 2. P53, EGF, PDGF, and NGF signalling pathways were upregulated in Gli4 with respect to GliT, while the axon guidance and TGF-β pathways as well as FA and cell adhesion genes were downregulated (Supplementary Table 1). In addition, quantification of the number of collective migration events per NS on NF +/− LN for Gli4 and GliT indicates that the shift from collective to single cell migration by the addition of LN is not as strong in GliT as in Gli4 (Fig. 5c,d). Apparently this collective behaviour is important for GliT cells as we found that their survival was compromised if they were seeded on NF as dissociated cells at low density (data not shown).

GliT also differs from Gli4 in the expression of proteins involved in the interaction with the ECM (Fig. 5d and Supplementary Tables 1, 2). For instance, galectin-3 protein levels decreased by 1.7 fold in GliT when plated on aNF + LN in comparison to 2D + LN (Fig. 5f and Supplementary Figure 2), whereas we found a 6-fold increase in the same conditions with Gli4. Integrin α6 expression in GliT increases on aNF +/− LN relative to 2D +/− LN (Fig. 5e and Supplementary Figure 2), whereas we observed a downregulation for Gli4 on NF + LN. Integrin β1 protein levels were not different between aNF +/− LN and 2D +/− LN for GliT cells (Fig. 5e and Supplementary Figure 2), whereas for Gli4 a higher integrin β1 protein expression was found on LN-coated NF than on uncoated NF. Immunofluorescence analysis of calpain-2 expression (Fig. 5f) shows equal expression of this enzyme in both 2D and on NF in GliT, contrary to Gli4. Also, GliT underexpress CAMK2D and overexpress CAMK2N1 with respect to Gli4 (Supplementary Table 2). CAMK2N1 is an inhibitor of the Ca^2+^/calmoduline–dependent protein kinase (CaMKII), which controls a range of cellular processes including cell adhesion and migration^27^. Altogether, our results show that Gli4 and GliT cells exhibit a different response of integrins -β1 -α6 and galectin-3 to ECM modifications.

### Integrin β1 and galectin-3 are upregulated in Gli4 cells in the CC whereas calpain-2 is downregulated in invasive Gli4 cells *in vivo*

**Figure 6:**
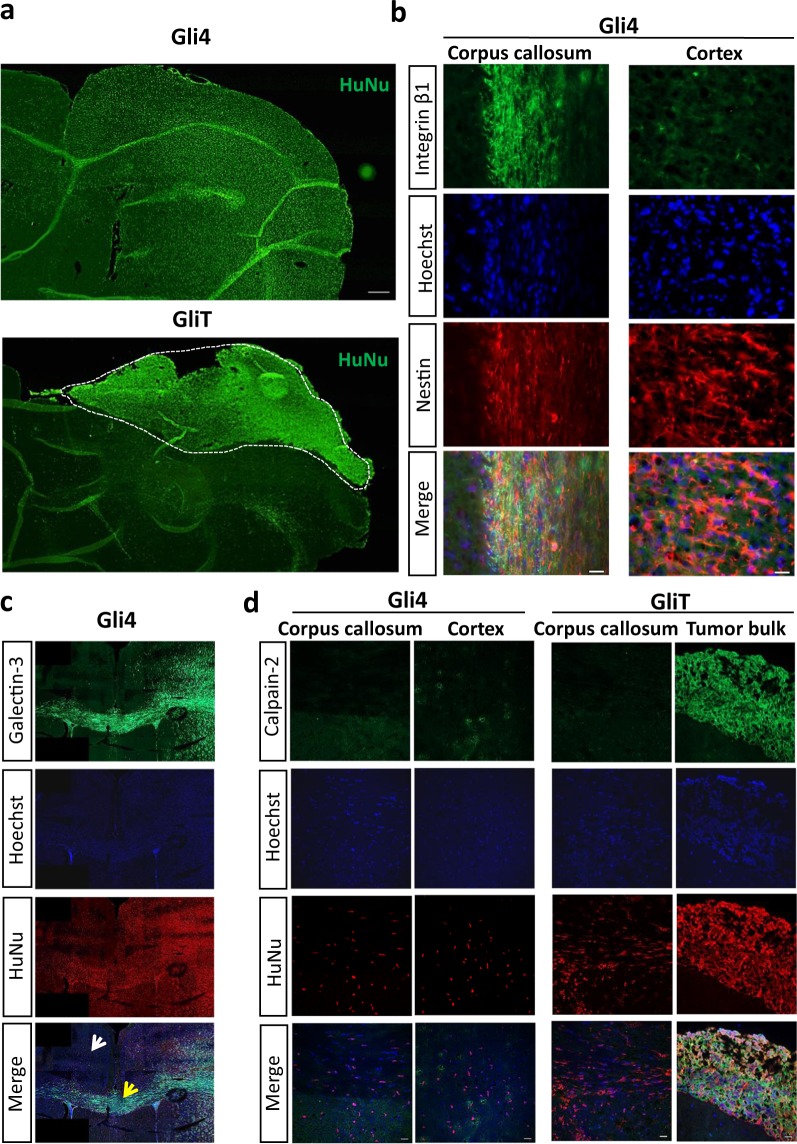
Integrin β1 and galectin-3 are upregulated in Gli4 cells migrating in the CC while calpain-2 is downregulated in invasive Gli4 and GliT cells. (**a**) Immunostaining for human nuclei (green) show the distribution of Gli4 and GliT cells in the mouse brain (scale bar = 300 µm). Gli4 cells are invasive, whereas GliT cells form a bulky tumour *in vivo* (white dotted area). (**b**) Immunostaining for integrin β1) and nestin of Gli4 cells. The images represent different areas of the brain (CC, cortex) (scale bar = 20 µm). (**c**) Immunostaining for galectin-3 (green) and human nuclei (red) of Gli4 cells migrating in the cortex (white arrow) and in the CC (yellow arrow). (**d**) Immunostaining for calpain-2 (green) and human nuclei (red) of Gli4 and GliT cells. Scale bar = 20 µm. Nuclei were stained with Hoechst 33342 (blue).

When transplanted in the brains of nude mice, Gli4 are highly invasive^20,28^ (Fig. 6a). They invade the cortex, proceed through the fibrous white matter tracts of the CC and reach the contra-lateral hemisphere (Fig. 6a). GliT cells in contrast, are almost not invasive and form a bulky tumour^28^ (Fig. 6a, area delineated by the dashed line). The expression of integrin β1 (Fig. 6b) and galectin-3 (Fig. 6c) in invasive Gli4 cells is higher when they migrate along the aligned myelinated fibres of the CC (Fig. 6c, yellow arrow) than in the cerebral cortex (white arrow) or the striatum. Calpain-2 is not expressed by invasive Gli4 cells in the CC nor in the cortex (Fig. 6d). Interestingly, GliT inside the tumour bulk express calpain-2 (Fig. 6d), while GliT migrating away from the tumour mass do not express calpain-2 (Fig. 6d). These data indicate that integrin β1 and galectin-3 expression depends on the cerebral microenvironment and determines invasiveness. Calpain-2 expression seems to be inversely correlated to the invasive potential of GIC *in vivo*.

## Discussion

After the stabilization step, FTIR analysis allowed us to determine that the proportion of pyridone (pyridine ring C_5_N bearing a carbonyl function) is very low in our compound given the absence of the carbonyl absorption band. The predominant functional groups (Fig. 1e) show that dehydrogenation and aromatization reactions occurred, in agreement with the colour and the fluorescence of the NF. The functional groups of the stabilized PAN NF promote hydrogen bond formation onto the cell surface, thus creating a biocompatible environment favouring cell attachment.

We have overcome a limiting problem encountered with electrospun NF consisting of the poor cellular infiltration or in-growth^3^. This is due to the small dimensions of the microenvironment which hamper the cells to insinuate their nucleus and cell body in the spaces separating adjacent NF. In the case of our PAN NF, alignment is not a requirement to create interstitial spaces permissive to cellular infiltration. This is likely due to the chemical nature, filament spacing and diameter of the material. These features allow the GICs to fully embed in a tridimensional microenvironment thereby abolishing the apico-basal polarity imposed by 2D. Also, our results show that the orientation of the NF governs the direction of migration of Gli4 cells much in the way that glioblastoma cells *in vivo* are guided by axon alignment in the CC. The addition of LN on aNF improves migration of Gli4 cells (Fig. 4b) as was reported previously for migration in the perivascular space *in vivo*^29^. The continuous actin cytoskeleton and N-Cadherin-mediated formation of cell-cell adherent junctions are responsible for the coordination between cells during collective Gli4 migration on uncoated NF (Fig. 3d). These cell-cell junctions at the leading edge as well as in lateral regions and inside the moving cell group are characteristic of collective migration^21^. Collective migration is a hallmark of epithelial cancer^30^, in contrast to the individual, mesenchymal, type of migration^31,32^. Similar to what has been described for ErbB2-positive breast cancer and lung tumour cells^33,34^, we propose that invading glioblastoma stem cells use collective migration to overcome anoikis.

Glioblastoma cells adapt outstandingly to various environments during their migration inside the brain. In our NF system, integrin β1 and galectin-3 overexpression coincides with a shift from collective to single cell migration (Figs 3 and 5). Unexpectedly, no difference in galectin 3 and integrin β1 mRNA content was discerned between 2D and NF conditions, even though a clear difference in protein levels was observed. This suggests that the regulation of expression of these proteins occurs after transcription^35,36^. Galectin 3 and integrin β1 overexpression in glioblastoma cells migrating along the white matter is typical of mesenchymal single cell migration^8,32^. Their overexpression by cells cultured on NF coated with LN and by cells migrating *in vivo* along the CC (Figs. 3 and 5) might indicate resistance to anoikis and a less proliferative (Fig. 4d), more metastatic phenotype^37^. Galectin-3 also acts as a multifunctional modulator of cell-cell and cell-ECM adhesion^38^, in particular promoting adhesion to LN^39^. The interaction of LN with Mgat5-modified N-glycans at the cell surface of mammary carcinoma cells stimulates α5β1-integrin activation and translocation to fibrillar adhesions^40^. Upregulation of the integrin β1 adhesion network at the plasma membrane as we observed with Gli4 cells grown on aNF + LN, not rarely goes hand in hand with a similar concerted raise in N-cadherin adherence (Fig. 3f)^41^. Both networks are mayor actors in to cell migration^42^, radio-resistance^43^ and immune-resistance^44^. The decrease of integrin-α6 in Gli4 cultured on NF + LN (Fig. 5a), is probably due to a loss of stemness of Gli4 cells in favour of a more invasive phenotype^45^. Our transcriptome analysis (Supplementary Figure 1) suggests that its role in heterodimer formation is taken by integrin α7.

While integrin β1, FAK and galectin 3 are overexpressed in cells grown on NF + LN in comparison to 2D + LN, neither an increase in calpain2 expression nor an increase in pFAK/FAK and talin/total talin ratios are observed when changing from NF –LN to + LN. These data indicate a stronger adhesion of Gli4 cells to the NF + LN independent of an increase of FA turnover. In 2D, LN promotes FA turnover without an increase of integrin β1, FAK and galectin 3 expression. In conclusion, the different responses (adhesion, turn-over and signalling) of Gli4 FA to LN are dependent on the physical properties of the support.

GliT maintains collective migration even on LN-coated NF (Fig. 5). This is in line with the observation that GliT migrate collectively in the CC, while Gli4 adopt a single cell mode (Fig. 6). We also found that calpain-2 is expressed by GliT cells staying inside the bulky tumour, while Gli4 cells migrating in the CC do not express calpain-2 (Fig. 6). With our NF model the expression of calpain-2 decreased for Gli4 grown on NF + LN in comparison to 2D + LN but not for GliT grown in the same conditions. Consistent with their maintenance of collective migration, GliT do not vary galectin 3 and integrin β1 expression in 2D and NF conditions. Moreover, Talin cleavage is not different in GliT grown in 2D NF +/− LN (Supplementary Figure 2). Calcium influxes are a unique characteristic increasing the invasiveness of glioblastoma cells. Calpain 2 is a key downstream effector of calcium ions, facilitating glioblastoma cell invasion^46^. As preliminary data, we recorded the occurrence of local calcium transients in filopodia of GliT cells collectively migrating on aNF + LN (Video 1). These transients are known to stabilise filopodia, but do not lead to a highly invasive phenotype. Also, according to our transcriptomic data, Gli4 and GliT in NS culture express various amounts of the ECM-related genes coding for LN, ECM receptor integrins and cell adhesion molecules (Supplementary Figure 2 and supplementary Table 2).

We propose that ECM attachment and sensing as well as the signal transduction pathways on which these depend are integrated in a different fashion in Gli4 than in GliT. It results in different migration modes, possibly due to different affinities to ECM proteins. Remarkably, all these different signal interactions sum up to complete different migrating behaviours of glioma cells *in vivo* i.e. (Fig. 6).

In conclusion, we have developed a new electrospun NF scaffold suitable for glioblastoma adhesion and migration in a 3D microenvironment akin mesenchymal migration. It allows for discrimination between the migration potential of different glioblastoma stem cells. The PAN NF matrix represents a valuable tool to study the role of the ECM in GIC migration. It can also serve as a relevant *in vitro* platform for compounds targeting PDL-1 whose expression is dependent on the interaction of FA with the ECM.

## Methods

### Cell culture

Gli4 and GliT were obtained as described by Guichet *et al*.^20^. Gli4 and GliT were cultured according to the NS protocol elaborated by Guichet *et al*.^20^. In DMEM/F12 medium supplemented with glucose, glutamine, insulin, N2, Epidermal Growth Factor and Fibroblast Growth Factor, (proliferation medium) GBM cells form NS, in DMEM/F12 medium supplemented with the same components as for the proliferation medium except from growth factors and heparin replaced by foetal bovine serum (0.5%), (differentiation medium) GBM cells migrate^20^. Prior to cell seeding, 2D or NF were either functionalized or not with poly-D-lysine added overnight^20^ and then the addition of LN (sigma L2020) (0.05 mg/mL) one hour at 37 °C. MTT test was used to evaluate cell viability as described^47^. To obtain NS of the same size, we used Corning® 96 well round bottom ultra-low attachment microplates coated with a covalently bond hydrogel (Corning 7007) (Supplementary Material 1). Dissociated GBM NS cultured in proliferation condition, were seeded in each well and remained in culture during 2 days until formation of single neurosphere due to sedimentation. Then GICs NS were deposited on the top of NF and left it to migrate during 5 days (Supplementary Material 1).

### NF production and characterization

aNF and naNF were produced by electrospinning using a solution of 10 wt% PAN (Aldrich, average Mw 150,000) dissolved in N,N-Dimethylformamide (DMF, Aldrich >99%, molecular biology grade). For the fabrication of the NF scaffold, a needle was used to project the polymer, which was collected on an electrode located 15 cm from the needle. A voltage of 20 kV was applied. To produce aNF, the collecting electrode was a rotating drum (2000 rpm), whereas a non-mobile metal disk was used to produce naNF. After electrospinning, the NF scaffold was thermally stabilized in a chamber furnace (250 °C, 2 h dwell, 2 °C. min^−1^ heating rate). ATR-FTIR spectra were recorded on a Perkin-Elmer Spectrum 100.

NF cryosectioning: For cryosectioning, the NF were included in OCT before freezing. Section thickness was 14 µm (Supplementary Material 2).

### GBM orthotopical xenotransplantation

Gli4 and GliT othotopic xenotransplantation were done as previously described^28^. After 3 months, the animals were sacrificed and brains were removed and post-fixed in 4% PFA and then immersed successively in 7%, 15% and 30% sucrose. Afterwards, the brains were included in an optimal cutting temperature (OCT) compound, snap-frozen and cryosectioned in 14 µm thick slices. All experiments involving animals were submitted to the local committee (division départementale de la protection des populations de l′Hérault) and approved under the Project Licence: C34-172-36. The lead experimenter holds a Level 1 Personal Licence under the reference I-34UnivMontp-F1-12.

### Immunofluorescence and 3D image reconstitution

Gli4 and GliT cultures were fixed by 4% PFA. Cell and brain sections were blocked and permeabilized using PBS - triton 0.5% - horse serum 5%. Primary antibodies were incubated overnight at 4 °C. The antibodies used in immunofluorescence were: N-Cadherin (abcam ab12221), Calpain-2 (abcam ab155666), Vinculin (Sigma Aldrich V9264), actin cytoskeleton was stained with Alexa Fluor 488 phalloidin (Molecular Probes A12379), cell nuclei with Hoechst 33342 (H3570 Life technologies) and Human Nuclei (Millipore MAB1281). Fluorochrome-coupled secondary antibodies were incubated 2 hours at room temperature (dilution 1/500). Image acquisition was realized using Zeiss Axioimager Z1/ Zen (with an apotome). Z-stack acquisition was realized using confocal microscopy (LSM 700). Imaris 8.1.2 software was used for image 3D reconstitution.

Migration quantifications were done using ZEN 2012 software in counting number of cells, using dapi staining, from 200 µm away from the border of the NS to the last observed migrating cell. Alternatively, migration capacity was quantified by measuring an area of migration. To measure migration areas, we subtracted the area of the NS containing non-migrating cells from the total area where cells were detected.

### Calcium imaging

After 6 days of migration, GliT were incubated before acquisition with 2 mM Fluo-4 AM (Invitrogen) and Pluronic (F-127) (Life technologies) in differentiation conditions. Time lapse calcium imaging in Z-Stack was performed using multiphoton microscopy during 45 min at 37 °C, 1 acquisition every 2 sec at 1% laser intensity. Imaris x64 8.1.2 software has been used for image 3D reconstitution.

### Scanning electron microscopy

Gli4 cells cultured on NF were fixed with 2.5% glutaraldehyde in PHEM buffer during 1 hour at room temperature then overnight at 4 °C. Cells were then dehydrated with successively 70%, 96% and 100% of alcohol and incubated in HMDS for drying.

### Western blot

Twenty µg of protein lysate were separated by SDS-PAGE. PVDF membranes were blocked by 0.1% TBS-Tween - 5% milk. Primary antibodies were incubated overnight at 4 °C. The antibodies used in western blot were: Galectin-3 (Abcam ab2785), Integrin β1 (Millipore AB1952), Integrin α6 (Abcam ab75737), FAK (Abcam ab40794), phospho-FAK Y397 (Abcam ab81298) Talin1/2 (Abcam ab11188), Calpain-2 (Abcam ab155666), Piezo 1 (Proteintech 15939-1-AP) and GAPDH (Millipore MAB374) as a loading control. Horseradish peroxidase-coupled secondary antibodies (Cell Signalling) were incubated 2 hours at room temperature. The Chemidoc XRS+ imager was used for chemiluminescence detection. Pixel quantifications were done with Image Lab software. Normalization by sum using total protein staining was used.

### Statistical analysis

The presented experiments were carried out at least in biological triplicates (except for the *in vivo* and the quantification of the number of migrating cells on aNF and naAF which were done twice). All the values are expressed as mean +/− SEM. Statistical tests were performed using the graphpad prism software.

### Transcriptome analysis

mRNA from glioblastoma lines was hybridised on Affymetrix Human Genome U133 plus 2.0 expression arrays by the Montpellier Institute for Regenerative Medicine and Biotherapy (https://www.mgx.cnrs.fr/) and Supplementary Material 3. Heatmaps were analysed with Serf software: http://bram.org/serf/Clusters.php. Overrepresentation analysis was carried out on-line with the Max Planck’s functional annotation page: http://cpdb.molgen.mpg.de

### Ethical approval and informed consent

I declare that all experimental protocols were approved by a named institutional and/or licensing committee.

I declare that all methods were carried out in accordance with relevant guidelines and regulations.

## Supplementary information

Local calcium transients in filopodia of GliT cells collectively migrating

Supplementary Information

## Data Availability

Data and associated protocols are promptly available to readers without undue qualifications in materials transfer agreements
