# Supplementary material for: A novel 3D nanofibre scaffold conserves the plasticity of glioblastoma stem cell invasion by regulating galectin-3 and integrin-β1 expression
Source: Sci Rep. 2019 Oct 10;9:14612. doi: 10.1038/s41598-019-51108-w (PMC6787018; doi:10.1038/s41598-019-51108-w)
Supplement: Supplementary file 2 — Supplementary Information [file 41598_2019_51108_MOESM2_ESM.pdf]

Supplementary Figure 1:

A novel 3D nanofibre scaffold conserves the plasticity of glioblastoma stem cell invasion by regulating galectin-3 and integrin-β1 expression. Ali Saleh, Emilie Marhuenda, Christine Fabre, Zahra Hassani, Jan de Weille, Hassan Boukhaddaoui, Sophie Guelfi, Igor Lima Maldonado, Jean-Philippe Hugnot, Hugues Duffau, Luc Bauchet, David Cornu and Norbert Bakalara

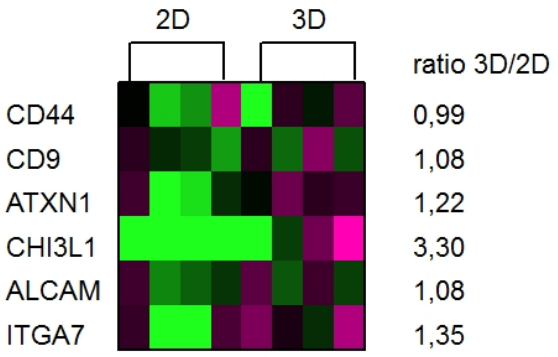

Heatmap of mesenchymal and pre-metastatic stem cell markers comparing expression in 2D and 3D conditions showing that GICs cultured in the 3D NF microenvironment retain their mesenchymal phenotype.

Supplementary figure 2: Western blot quantification of Gli4 and GliT.

A novel 3D nanofibre scaffold conserves the plasticity of glioblastoma stem cell invasion by regulating galectin-3 and integrin-β1 expression. Ali Saleh, Emilie Marhuenda, Christine Fabre, Zahra Hassani, Jan de Weille, Hassan Boukhaddaoui, Sophie Guelfi, Igor Lima Maldonado, Jean-Philippe Hugnot, Hugues Duffau, Luc Bauchet, David Cornu and Norbert Bakalara

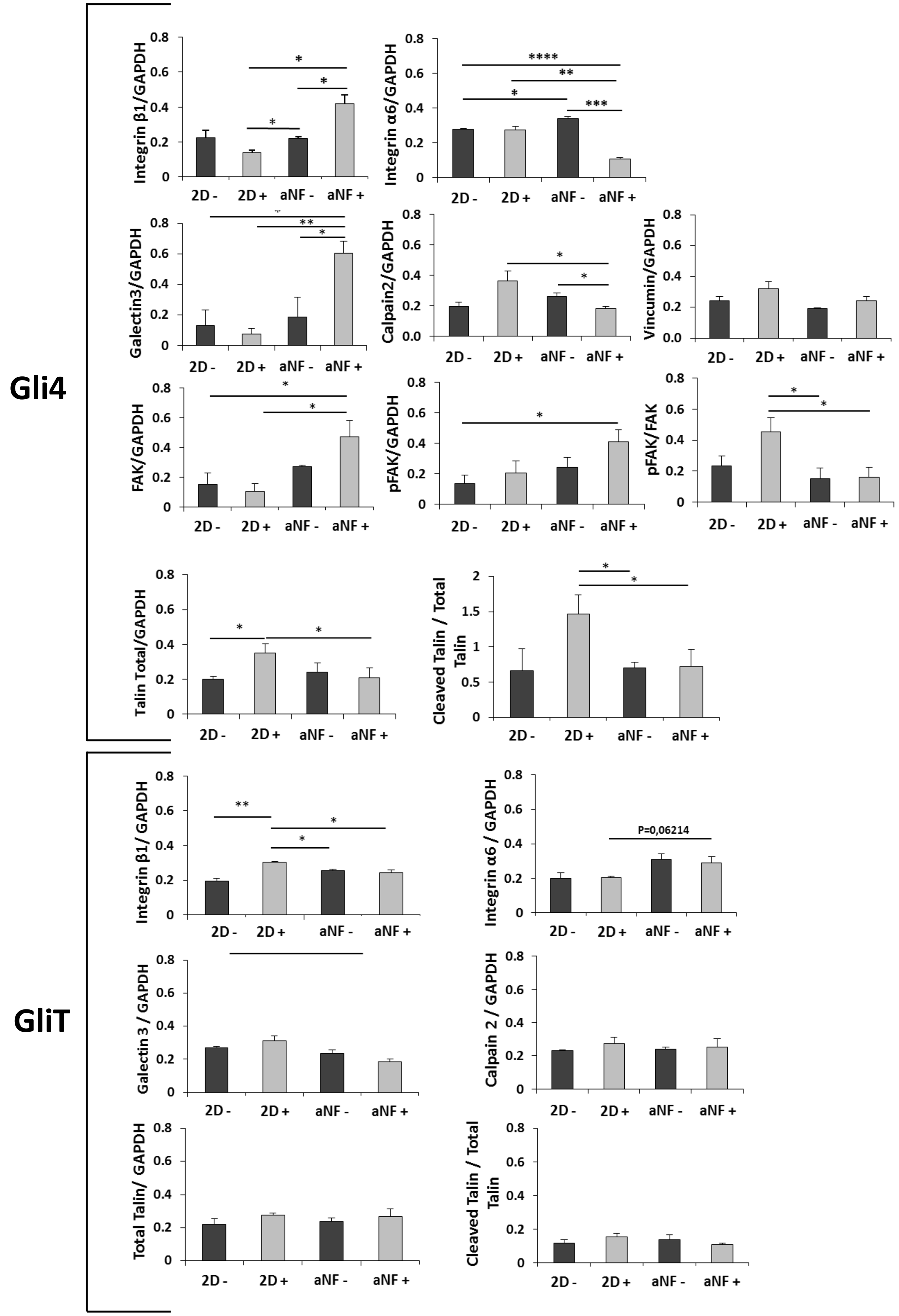

Western blot quantification of Gli4 and GliT. Each western blot quantification was performed three times and normalized with respect to GAPDH expressions. Bands were quantified using the Bio-Rad Chemidoc and the image lab software . Data are presented as mean +/- SEM.

Supplementary Material 1: Control of the cell number and the volume of Gli4 neurospheres.

A novel 3D nanofibre scaffold conserves the plasticity of glioblastoma stem cell invasion by regulating galectin-3 and integrin-β1 expression. Ali Saleh, Emilie Marhuenda, Christine Fabre, Zahra Hassani, Jan de Weille, Hassan Boukhaddaoui, Sophie Guelfi, Igor Lima Maldonado, Jean-Philippe Hugnot, Hugues Duffau, Luc Bauchet, David Cornu and Norbert Bakalara

A

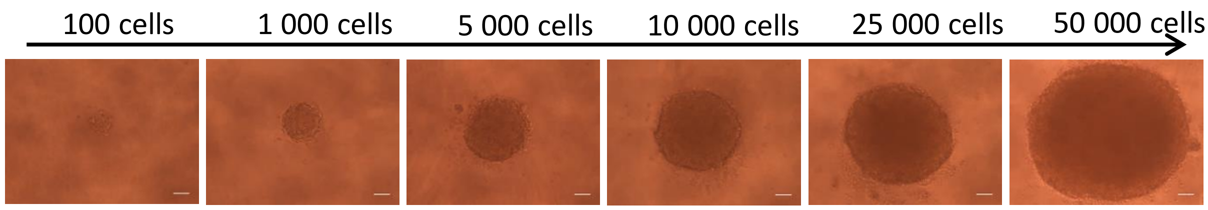

B

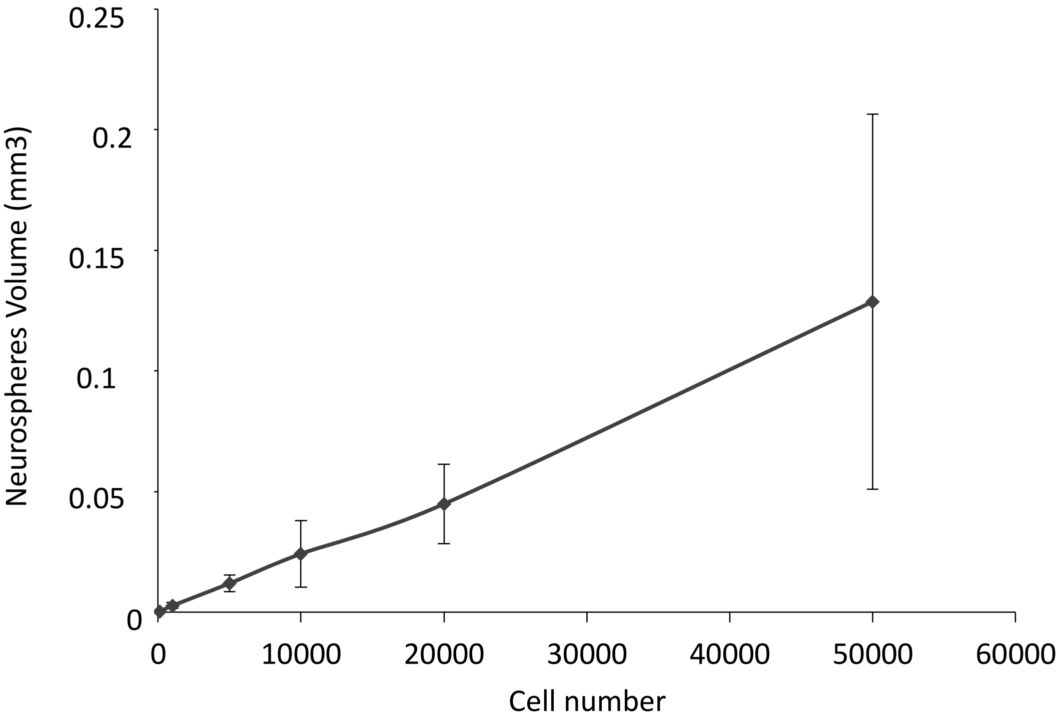

Gli4NS containing the same number of cells and volume were obtained using CorningTM ultra low attachment microplates coated with a covalently bonded hydrogel . (A)Images of the NS formed by 100 to 50,000 cells, captured 24 hours after seeding. Each well contains only one NS. (B)NS volume (mm3) as a function of cell number after 24 hours of seeding (n=12).

Supplementary Material 2: Neurosphere deposit and cryosectioning

A novel 3D nanofibre scaffold conserves the plasticity of glioblastoma stem cell invasion by regulating galectin-3 and integrin-β1 expression. Ali Saleh, Emilie Marhuenda, Christine Fabre, Zahra Hassani, Jan de Weille, Hassan Boukhaddaoui, Sophie Guelfi, Igor Lima Maldonado, Jean-Philippe Hugnot, Hugues Duffau, Luc Bauchet, David Cornu and Norbert Bakalara

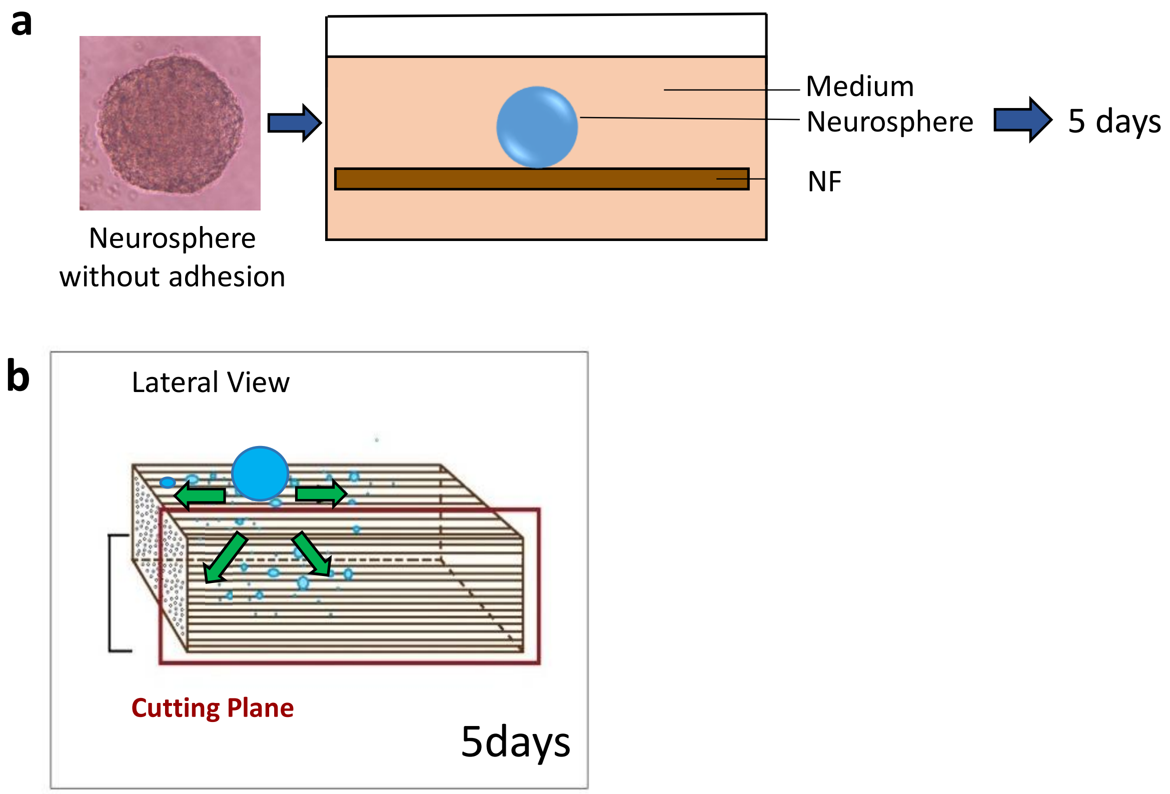

a. GICs neurospheres were deposited on the top of the matrix to which they will adhere, we supply them with migration medium and leave it to migrate during 5 days. The same method was used for dissociated cells. **b.** On Fig 2.b nanofiber felts were fixed, included in OCT before freezing and then we performed cutting planes by cryosectioning , on the transversal plane of the nanofibrers matrix, in order to observe cell migration inside the felt.

Supplementary Material 3: Transcriptomic analysis protocol:

A novel 3D nanofibre scaffold conserves the plasticity of glioblastoma stem cell invasion by regulating galectin-3 and integrin-β1 expression. Ali Saleh, Emilie Marhuenda, Christine Fabre, Zahra Hassani, Jan de Weille, Hassan Boukhaddaoui, Sophie Guelfi, Igor Lima Maldonado, Jean-Philippe Hugnot, Hugues Duffau, Luc Bauchet, David Cornu and Norbert Bakalara

**label protocol**	Biotinylated cRNA were prepared according to the Affymetrix GeneAtlas IVT PLUS protocol from 100 ng total RNA (GeneAtlas 3'IVT PLUS Technical Manual, 2013-2015, P/N703210 Rev2 Affymetrix).
**hyb protocol**	Following fragmentation, 10 ug of cRNA were hybridized for 16 hr at 45°C on the Affymetrix® U133+PM Array.
**wash protocol**	StripChips were washed and stained on the fluidic station of the Affymetrix GeneAtlas system using Hybridization Wash & Stain kit.
**scan protocol**	Microarrays were scanned using the Affymetrix GeneAtlas scanner.
**data processing**	Data were generated with Affymetrix Expression Console v 1.4.1 and GCRMA algorithm.<br>Heatmaps were analysed with Serf software http://www.bram.org/serf/Clusters.php. Overrepresentation analysis was carried out on-line using the Max Planck Institute's functional annotation page: http://cpdb.molgen.mpg.de/

Each of the experimental conditions was carried out in duplicate and hybridized on two separate GeneChips. By creating a frequency histogram of all expression levels obtained with the Gli4 and GliT lines, two populations of probes were found: those who bound and those who did not bind mRNA from these cell lines. A particular gene was supposed to be expressed if the expression value exceeded 56. The ratio of gene expression of pairs of experimental conditions was calculated by first averaging the expression levels on the two chips per condition followed by taking the ratio of the two averages. Ratios between 0.5 and 2 were considered to be insignificant. Heatmaps were analysed with Serf software http://www.bram. org/serf/Clusters.php. Overrepresentation analysis was carried out on-line with the Max Planck Institute's functional annotation page: http://cpdb.molgen.mpg.de/.

Supplementary Table 1: Transcriptome analysis of Gli4 and GliT cells.

A novel 3D nanofibre scaffold conserves the plasticity of glioblastoma stem cell invasion by regulating galectin-3 and integrin-β1 expression. Ali Saleh, Emilie Marhuenda, Christine Fabre, Zahra Hassani, Jan de Weille, Hassan Boukhaddaoui, Sophie Guelfi, Igor Lima Maldonado, Jean-Philippe Hugnot, Hugues Duffau, Luc Bauchet, David Cornu and Norbert Bakalara

Pathways downregulated in Gli4		Pathways upregulated in Gli4	
**Name**	p-value	**Name**	p-value
**Axon guidance**	$1.53x10^{-6}$	**P53 signalling**	$3.81x10^{-7}$
**L1CAM interactions**	$1.38x10^{-5}$	**EGFR signalling**	$4.55x10^{-6}$
**TGF-beta signalling**	$1.16x10^{-4}$	**EGFR signalling**	$7.76\ x10^{-6}$
**Cell adhesion**	$2.26x10^{-4}$	**NGF signalling**	$3.02x10^{-5}$
**Focal adhesion**	$2.31\ x10^{-4}$	**SCF/KIT signalling**	$7.16x10^{-5}$

List of selected pathways differentially expressed in Gli4 and GliT cultured as NS in proliferation medium

Supplementary Table 2: Transcriptome analysis of Gli4 and GliT.

A novel 3D nanofibre scaffold conserves the plasticity of glioblastoma stem cell invasion by regulating galectin-3 and integrin-β1 expression. Ali Saleh, Emilie Marhuenda, Christine Fabre, Zahra Hassani, Jan de Weille, Hassan Boukhaddaoui, Sophie Guelfi, Igor Lima Maldonado, Jean-Philippe Hugnot, Hugues Duffau, Luc Bauchet, David Cornu and Norbert Bakalara

Gene Name	Expression Ratio Gli4/GliT	P_value
**LAMA2**	4.93	0.05
**LAMA1**	0.146	0.046
**COL11A1**	5.125-6.348-7.07	0.016-0.011-0.013
**COL4A6**	0.269	0.002
**COL20A1**	0.332	0.023
**COL22A1**	0.41	0.008
**ITGA10**	2.671	0.04
**ITGA6**	0.48	0.00044
**ITGB5**	0.384	0.007
**L1CAM**	0.032	0.00023
**CAMK2N1**	0.087-0.187	0.00048-0.008
**MCAM**	0.351	0.00050
**NRCAM**	0.411	0.031
**CAMK2D**	4.568	0.031

List of ECM and cell adhesions genes differentially expressed in Gli4 and GliT cultured in proliferation medium
